# Optical cavity-mediated exciton dynamics in photosynthetic light harvesting 2 complexes

**DOI:** 10.1038/s41467-022-34613-x

**Published:** 2022-11-11

**Authors:** Fan Wu, Daniel Finkelstein-Shapiro, Mao Wang, Ilmari Rosenkampff, Arkady Yartsev, Torbjörn Pascher, Tu C. Nguyen- Phan, Richard Cogdell, Karl Börjesson, Tönu Pullerits

**Affiliations:** 1 Division of Chemical Physics and NanoLund, Lund University, Lund, Sweden; 2 Instituto de Química, Universidad Nacional Autónoma de México, Mexico, Mexico; 3 Department of Chemistry and Molecular Biology, University of Gothenburg, Gothenburg, Sweden; 4 School of Molecular Biosciences, University of Glasgow, Glasgow, UK

**Keywords:** Chemical physics, Kinetics, Biophotonics

## Abstract

Strong light-matter interaction leads to the formation of hybrid polariton states and alters the photophysical dynamics of organic materials and biological systems without modifying their chemical structure. Here, we experimentally investigated a well-known photosynthetic protein, light harvesting 2 complexes (LH2) from purple bacteria under strong coupling with the light mode of a Fabry-Perot optical microcavity. Using femtosecond pump probe spectroscopy, we analyzed the polariton dynamics of the strongly coupled system and observed a significant prolongation of the excited state lifetime compared with the bare exciton, which can be explained in terms of the exciton reservoir model. Our findings indicate the potential of tuning the dynamic of the whole photosynthetic unit, which contains several light harvesting complexes and reaction centers, with the help of strong exciton-photon coupling, and opening the discussion about possible design strategies of artificial photosynthetic devices.

## Abstract (web summary)

Light-matter interaction can induce changes to the properties of the system by creating hybrid collective states of light and molecular excitations, the so called polaritons. Here the authors use femtosecond pump-probe spectroscopy to explore exciton-polariton dynamics in a photosynthetic protein, light harvesting 2 complexes, and find evidence for rapid energy transfer to dark polariton states.

## Introduction

There has been growing interest in light-matter interactions in the past decades not only because the photo-induced reactions and the related applications, but also due to the ability to alter the intrinsic properties of the matter^1–5^. Optical cavities have been demonstrated to be a powerful tool to mediate and control the light matter interactions. When the energy exchange rate between the molecules assembled in the cavity and the localized electromagnetic field is faster than the dephasing rate of the molecular transitions and photons, strong coupling between molecules and the cavity is achieved. This strong coupling creates two new optically allowed hybrid light–matter states, which have energies different from the molecular electronic transitions and the cavity mode, and are termed as the upper polariton state (UP) and lower polariton state (LP). In case of resonance between the cavity mode and the molecular transition, the energy separation between the UP state and LP state is the Rabi splitting (ħΩ_R_). This can be experimentally characterized as the peak splitting around the exciton energy in steady-state absorption spectrum of the coupled system. The strong light-matter coupling can also modify the energy transfer process^6–8^, photochemical reactions^9–11^, conductivity^12^, and charge carrier mobilities^13^, among others. Strong coupling between organic molecules and cavities has been achieved on J-aggregates^14–16^, organic semiconductors^6,7^, and photosynthetic systems^17–19^. Strong coupling can be achieved also by using plasmonic effects^19,20^. For example, it has been recently shown that one of the photosynthetic proteins, light harvesting 2 (LH2) complex can be strongly coupled to a plasmonic nanostructure, thereby modifying the optical properties of LH2 antennas^19^. Experimental evidences of strong coupling in ensembles of chlorosomes and living bacteria with optical cavities have been reported^17,18^. However, the underlying polariton dynamics involving photosynthetic systems has been limited to theoretical studies^21,22^. In this article, we demonstrate the experiment using transient absorption spectroscopy to reveal the polariton dynamics of LH2 complex from purple bacteria *Rhodoblastus acidophilus*, confined in a Fabry−Pérot (FP) optical microcavity.

**Fig. 1:**
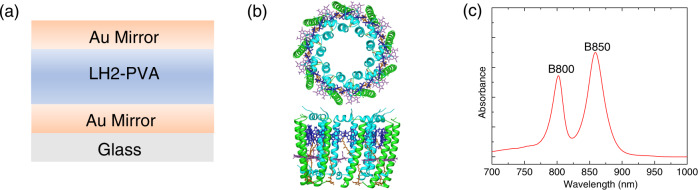
Microcavity structure and LH2 properties. **a** Structure of the semi-transparent Fabry–Pérot cavity, where two Au mirrors (thickness of 22 nm) sandwich a PVA film containing LH2 (thickness of 300 nm); **b** structure of LH2 complex from *Rhodoblastus acidophilus* strain 10050 with a view normal to the membrane plane (top) and a side view of the complex (down), where the closely packed B850 ring is shown in blue, the loosely packed B800 ring in mauve, the carotenoid chains in brown, the α-apoprotein chains in cyan and the β-apoprotein chains in green; **c** steady state absorption of LH2 bare film on glass substrate, where the B800 and B850 absorption peaks are well resolved.

LH2 complexes form the peripheral light harvesting antenna of the photosynthetic purple bacteria. They absorb solar energy forming electronic excitations which are transferred to the core light harvesting complex 1 and further to the reaction center where charge separation takes place driving the whole process of photosynthesis. In the LH2 of *Rhodoblastus acidophilus*, there are two rings of bacteriochlorophyll (BChl) *a* molecules embedded into a protein scaffold^23^ (see Fig. 1). One ring consists of nine weakly interacting BChl *a* molecules, absorbing at 800 nm and thereby called B800, whereas the other ring consists of 18 closely packed BChl *a* molecules with significant excitonic interaction, absorbing around 850 nm, called B850. Such well resolved spectral and characteristic structural features have stimulated numerous studies of the optical properties and the exciton dynamics in LH2^24–29^. The intra-LH2 energy transfer from B800 to B850 is reported to be ~1 ps and the excited state lifetime in B850* is about ~1 ns. In the LH2 aggregates, the energy transfer time between different complexes is ~5 ps. These spectral and dynamic properties in LH2 are well described and understood, making the complex an ideal model system for studies of spectroscopy and dynamics in strongly coupled biological polariton system.

In this work, we experimentally demonstrate strong exciton-photon coupling between a confined cavity mode and the low energy electronic transition of the LH2 complexes. To realize this, a Fabry–Pérot (FP) cavity consisting of two parallel gold mirrors, sandwiching a polyvinyl alcohol (PVA) film containing LH2s was constructed. To verify the strong coupling regime, the dispersive behavior of the absorption of the cavity was inspected and showed a clear anti-crossing around the exciton transition energies. Significantly, we have observed substantial changes in exciton dynamics due to the creation of polariton states in LH2. By means of ultrafast pump probe spectroscopy, the kinetics probed at several different polariton energies validate the formation of the polariton states and reveal a longer lifetime of the strongly coupled system compared with the bare exciton.

## Results and Discussion

### Microcavity structure and LH2 properties

The structure of the used microcavities is shown schematically in Fig. 1a. The cavity consists of a 300 nm thick layer of LH2 dispersed in a polyvinyl alcohol (PVA) matrix sandwiched between two 22 nm thick semi-transparent Au mirrors, resulting in a *λ*/2 cavity. Au was selected as the mirror material because of its high stability in air and high reflectivity in the wavelength region above 600 nm compared with Al and Ag. Figure 1c shows the steady-state absorption spectrum of a control film of LH2 in PVA. The absorption bands of LH2 are visible as peaks at 801 nm and 860 nm, corresponding to the B800 and B850 bands, respectively. The Q-factor of the cavity was determined from a negatively detuned LH2 containing cavity sample where the normal cavity mode is red-shifted compared with the exciton states of LH2. The Q-factor for this cavity is approximately 15 (Supplementary Fig. 1), which is in agreement with the reported Q-factors for similar optical cavity systems^30^.

### Angle-resolved steady-state absorption measurements

**Fig. 2:**
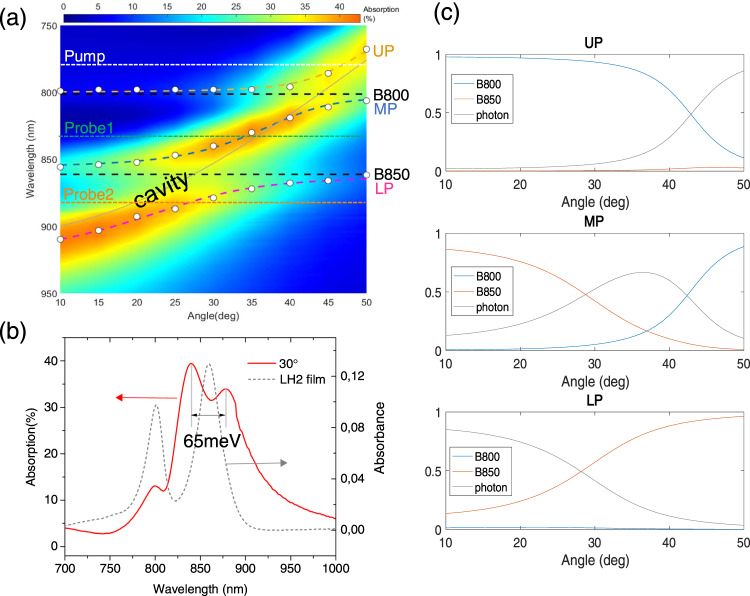
Rabi splitting in LH2 polaritons. **a** Angle-resolved absorption (transverse electric mode) spectra and the fitting results from a 3-by-3 coupled oscillator model; **b** absorption spectrum of the strong coupled cavity at 30° (red solid line) and the LH2 film absorbance (dashed gray line) **c** the Hopfield coefficients of each polariton state.

To evaluate the possible strong coupling in the cavity containing LH2, angle-resolved reflection and transmission spectra were measured (see Supplementary Fig. 2) and corresponding angle-resolved absorption spectra were extracted as *A* = 1-*T-R*, as shown in Fig. 2a. From the dispersion curve of this cavity sample, three polariton branches can be resolved as the upper polariton branch (UP), the middle polariton branch (MP) and the lower polariton branch (LP). The energy of the polariton branches was well fitted with the coupled oscillator model (see the Methods Section). The Rabi splitting energy of B850 band and B800 band was fitted to 61 meV and 47 meV, respectively. The fitted polariton branch positions are shown as yellow (UP), blue (MP), and pink (LP) dashed lines, with the photon mode and exciton wavelengths shown with gray solid line and black dashed lines, respectively. A clear anti-crossing behavior around the B850 band can be seen in the dispersion curve, indicating the achievement of strong coupling. Figure 2b shows the cavity absorption spectrum at the angle (30°) where the cavity mode and the B850 exciton are close to resonance. An obvious peak splitting at around 860 nm with a separation of 65 meV is detected. The strong coupling can be also assessed by using criterion^2,18^ *ħΩ*_R_ ≥ (*γ*_M_ + *γ*_C_)/2, where *ħΩ*_R_ is the Rabi splitting energy, *γ*_M_ and *γ*_C_ are the full width at half maximum linewidths of the bare molecule exciton (*γ*_M_) and the cavity mode (*γ*_C_), respectively. The linewidth of the B850 band was determined to be 42 meV from the steady state absorption spectrum. The linewidth of the cavity was obtained from transmission spectrum of a negatively detuned cavity, which is around 78 meV (see Supplementary Fig. 1). Indeed, the Rabi splitting energy of the B850 band (61 meV) is larger than (*γ*_M_B850_ + *γ*_C_)/2 = 60 meV, confirming that the B850 band is strongly coupled to the cavity. Strictly, the system is on the edge of the strong coupling. By contrast, at the peak of B800 band, only a faint peak splitting with milder anti-crossing takes place. The linewidth of the B800 band was likewise determined from the steady state absorption spectrum to be 40 meV. The comparison of the Rabi splitting energy (46 meV) with the value of (*γ*_M_B800_ + *γ*_C_)/2 = 59 meV indicates that the B800 band is in the weak or intermediate coupling regime. The difference between the two splittings, which is directly related to the coupling strength of the two excitonic bands with the cavity, can be explained by the different number of BChl a molecules contributing to the B850 ring and the B800 ring in LH2 considering that the coupling strength is proportional to the square root of the concentration of the molecules inside the cavity^31^. Figure 2c displayed excitonic and photonic mixing coefficients of the polariton branches. We can see that MP and LP consist of almost equal contributions from B850 band and the cavity photon at around 30°. Similarly, MP and LP possess almost the same ratios of B800 band and the photon near 45°.

### Femtosecond pump probe measurements

**Fig. 3:**
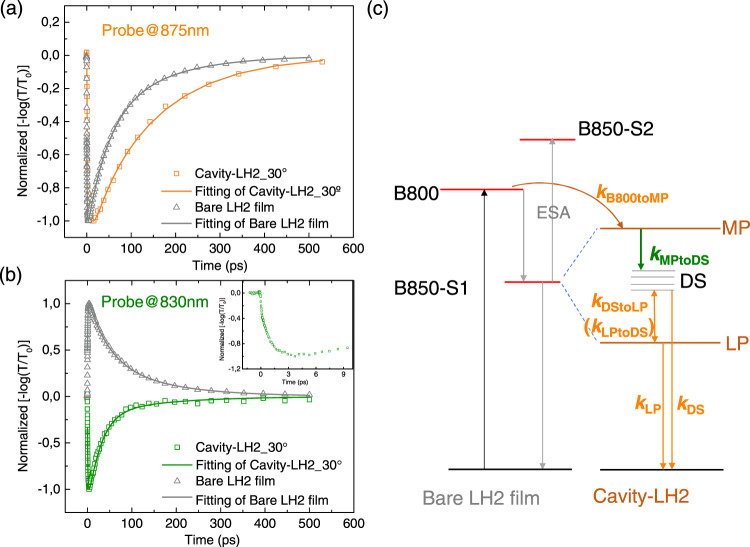
LH2 polariton dynamics. Pump probe kinetics (scattered markers) of bare LH2 film and angled cavity at 30° with pump excitation at 785 nm (8.5 uJ/pulse/cm^2^) and probe at **a** 875 nm and **b** 830 nm (the inset shows the kinetic signal in the first 10 ps) and corresponding **c** energy relaxation pathways. The solid lines are the fitting results based on the rate-based kinetic models.

To gain further insight, the excitation dynamics of the system was analyzed with femtosecond pump probe spectroscopy. The broadband pump probe spectra of bare LH2 film and strongly coupled cavity sample are shown in Supplementary Fig. 4, where clear spectral differences are demonstrated. Prior to any further analysis of the pump probe data of the polaritonic system, it is of great importance to exclude any non-polaritonic effects induced by the pump excitation, e.g. Rabi contraction, thermal expansion of the cavity, and bulk refractive index changes, which have been extensively analyzed by Musser and coworkers^32^. Herein, we calculate the spectra from all the non-specific photoexcitation induced effects with the 3-by-3 coupled oscillator model (see Supplementary Note 2 and Supplementary Fig. 5), which are obviously deviating from the experimental pump probe spectra of the strongly coupled cavity sample, indicating that the pump probe signals measured in this work mainly correspond to the true dynamics of the polaritons. In addition, it is also worth noting the effect of the uncoupled molecules in the cavity on the pump probe signals. These include the molecules which are outside the photonic-mode volume and the molecules whose transition dipole moment is orthogonal to the cavity mode polarization. Contribution by such uncoupled molecules has recently been discussed in vibrational polariton systems by Xiang et al.^33^ and Duan et al.^34^. Herein, to evaluate the potential contribution from the uncoupled molecules, a weakly coupled LH2 containing cavity sample which shows no obvious Rabi splitting in the steady-state transmission spectrum (see Supplementary Fig. 4b) was prepared and studied with ultrafast pump probes spectroscopy. The corresponding pump probe spectrum is shown in Supplementary Fig. 4a. Comparing the pump probe spectra of the strongly coupled cavity sample with the weakly coupled sample and the bare LH2 film at 1–2 ps (see supplementary Fig. 4a), we can see that the spectrum of the weakly coupled sample is very similar to the spectrum of the bare LH2 film, while the spectrum of the strongly coupled sample differs significantly from the other two. Clearly, the polaritons provide major contribution to the spectra in case of strong coupling thereby even the measured ultrafast dynamics mainly corresponds to the dynamics of the polariton excitations. Figure 3 compares the pump probe kinetics of the LH2 film outside the cavity with that of the coupled system. The pump wavelength was set to be 785 nm, exciting the blue edge of the B800 band. The laser pulses (both pump and probe) are placed at 30° with respect to the normal direction to the cavity surface, where the cavity energy was resonant with the B850 exciton. From the corresponding absorption spectrum, as shown in Fig. 2b, we can clearly see three absorption peaks at 801 nm, 840 nm and 879 nm, which represent the B800 band, the MP state and LP state resulting from the strong coupling of B850 band with the cavity, respectively. Two different probe wavelengths were selected accordingly at 830 nm and 875 nm, which were close to the MP state and LP state energy. Figure 3a presents the kinetics probed at 875 nm, where both the LP state of the cavity sample and red tail of the bare B850 band reside. Ground-state bleach signals from both the B850 band of the bare LH2 film (gray line) and the LP state of the strongly coupled system (orange line) were observed. We note that the lifetime of the strongly coupled system is much longer than the bare LH2 film. Similar behavior has been reported previously in other organic polariton systems and explained by the exciton reservoir theory^2,35–37^. The possible energy relaxation pathway was displayed in Fig. 3c, which was assumed to be modified by the strong-coupling created polaritonic states and dark states (DS). The corresponding dynamic process was semi-quantified using a rate-based kinetic model^38,39^, where we minimized the possible effect from the transient shift of the ground-state absorbance to simplify the fitting. The rate equations can be written as below:

1 $$\frac{d[{B800}^{*}]}{{dt}}=-{k}_{{{\mbox{B}}}800{{\mbox{toMP}}}}\left[{B800}^{*}\right]$$

2 $$\frac{d[{MP}]}{{dt}}={k}_{{{\mbox{B}}}800{{\mbox{toMP}}}}\left[{B800}^{*}\right]-{k}_{{{\mbox{MPtoDS}}}}\left[{MP}\right]$$

3 $$\frac{d[{DS}]}{{dt}}={k}_{{{\mbox{MPtoDS}}}}\left[{MP}\right]-{k}_{{{\mbox{DStoLP}}}}\left[{DS}\right]+{k}_{{{\mbox{LPtoDS}}}}\left[{LP}\right]-{k}_{{{\mbox{DS}}}}\left[{DS}\right]$$

4 $$\frac{d[{LP}]}{{dt}}={k}_{{{\mbox{DStoLP}}}}\left[{DS}\right]-{k}_{{{\mbox{LPtoDS}}}}\left[{LP}\right]-{k}_{{{\mbox{LP}}}}\left[{LP}\right]$$

Here *k*_B800toMP_ is the energy transfer rate from the excited B800 band to the MP state, *k*_MPtoDS_ represents the rate of energy transfer from the MP state to the dark states, *k*_DStoLP_, (*k*_LPtoDS_) refers to the rate of energy transfer from dark states to the LP state (and vice versa), and *k*_DS_ and *k*_LP_ is the rate of dark states and LP decaying to the ground state, respectively. Table 1 summarized one set of numerical values of the parameters obtained from the fitting procedure. After initial excitation of the B800 band, the energy is relaxed to the MP with a rate constant of ~ 1 ps^−1^, which agrees with the rate of energy transfer from the B800 band to the B850 band in LH2 solution. From there, the excited state energy is further relaxed down to the dark states of the exciton-polariton system and then to the lower polariton state. According to Fermi’s golden rule, the transition rate is proportional to the density of the final states. Since density of dark states can be very high, the relaxation from the MP to the dark state manifold will be fast compared with the process from the dark states to LP. This is also validated by the modeling results, where the former rate is faster than the latter rate. Moreover, it has recently been argued that owing to the entropy contribution originating from the high number of the dark states, the free energy of these states can be even lower than the LP^40^, making the transition from LP to dark states another possible energy relaxation pathway. Accordingly, the decay of the populated dark states determines the lifetime of the whole system, as shown in Table 1 with the slowest decay rate constant of 0.0041 ps^−1^. Since the decay of the dark states only possesses the nonradiative channel as contrasted with the decay of the bare exciton which conducts both radiative and nonradiative pathways, it is expected to observe a longer lifetime of the strongly coupled system than the uncoupled bare exciton. Analogously, the interplay between the polariton states and the dark states has also been established in the strongly coupled vibrational polariton systems^33,34,41^.

**Table 1:** Results of modeling for the strong coupled system

Parameter	Rate constant (ps^−1^)
*k*_B800toMP_	0.98
*k*_MPtoDS_	0.13
^*^k_DStoLP_	0.007 ± 0.002
^*^k_LPtoDS_	0.016 ± 0.005
^*^k_DS_	0.0041 ± 0.0008
^*^k_LP_	0.016 ± 0.003

^*^The numerical values for these parameters are not exclusive from the fitting, but with uncertainties. Fitting plots with uncertainties of *k*_DStoLP_, *k*_LPtoDS_, *k*_DS_, and *k*_LP_ are shown in Supplementary Fig. 6.

The pump probe kinetics probed at 830 nm for both the bare LH2 film and the strongly coupled cavity samples are presented in Fig. 3b. For the bare LH2 film, the positive signal corresponds to an excited state absorption (ESA) consistent with previous analogous studies^25,42^. In contrast, a negative signal was observed for the strongly coupled system due to the Rabi splitting contraction around the MP region^30^, which indirectly validates the formation of polariton states. The inset shows a rising kinetic signal in the first few ps, which represents the energy transfer process from excited B800 band to the MP state. Comparing the kinetics of the two cases (see Supplementary Fig. 7), we observe that in the strongly coupled system the decay is faster than in the bare LH2 film. We assign the faster decay in the cavity system to the energy transfer from the MP to the dark states. As discussed above, based on the density of states argument, this transfer step is expected to be faster than the decay of the uncoupled B850 excited states.

In conclusion, we have demonstrated strong coupling between a low-Q Fabry−Pérot metallic optical cavity and the B850 exciton band of the LH2 from purple bacteria with a Rabi splitting of 61 meV for the first time. Pump probe spectroscopy has revealed significantly different exciton dynamics in the strongly coupled system, compared with the uncoupled LH2 film. When probing at 830 nm, the negative signal is detected in the strongly coupled system in contrast to the positive ESA signal of the uncoupled LH2 film. The observation is explained by the Rabi contraction consistent with the formation of the polariton states. Besides, the strongly coupled system showed a much longer lifetime than the bare LH2 film, which affirms the important role of the dark states to the energy transfer. Since polariton state can form an efficient energy relaxation pathway between spatially and energetically separated exciton species, further exploration of the possible cavity mediated energy transfer between LH2s and to the RC would be of great interest.

## Methods

### Sample preparation

LH2 complexes were isolated from *Rhodoblastus acidophilus10050* as reported previously^43^ and dispersed in a TL buffer (0.1% LDAO, 20 mM Tris.HCl pH 8.0) and stored at −80 °C as a stock solution. The optical cavities were built on glass substrates (15 × 15 mm^2^), which were cleaned by successive sonication in alkaline solution (0.5% of Hellmanex in deionized water, 15 min), deionized water (15 min), acetone (15 min) and isopropanol (15 min), followed by an oxygen plasma treatment for 1 min. Subsequently, a semi-transparent Au mirror with a thickness of 22 nm was deposited on the glass substrate by vacuum sputtering deposition (AJA Orion 5). To prepare the active polymer layer, the PVA was dissolved in the above-mentioned Tris buffer at a concentration of 70 mg/ml. Then LH2 stock solution was mixed with the aqueous PVA solution at a volume ratio of 5:3 with a vortex mixer, and passed through a PVDF filter (0.45 mm pore size). Oxygen scavengers were added to the mixed solution to protect LH2 from photooxidation^44,45^. The mixed solution was spin-coated (Laurell Technologies WS-650) onto the Au mirror coated glass substrate at a speed of 1500 rpm for 1 min. Thereafter, a second Au mirror of 22 nm was deposited on top of the polymer layer by vacuum sputtering deposition. The fabricated cavity was kept under vacuum in the dark at −20 °C to avoid any oxidation and any aging of the sample. For comparison, a reference sample of bare LH2 film was fabricated using the same mixed solution and parameters for spin-coating on a clean glass substrate without the Au mirrors.

### Steady-state spectroscopy

All the steady-state spectra were measured using a standard spectrophotometer (Lambda 950, Perkin Elmer) with accessories. The angle-resolved transmission spectra were recorded having a variable angle accessory. A universal reflectance accessory was used to obtain the angle-resolved reflection spectra.

### Femtosecond pump probe spectroscopy

Ultrafast pump probe measurements were performed on two in-house built setups for single-color probe and broadband probe detections, respectively. The broadband femtosecond pump probe measurements were carried out based on a Solstice (Spectra Physics) amplified laser system that produces ~60 fs pulses at a central wavelength of 796 nm at 4 kHz repetition rate. The laser output is split into two parts to generate pump and probe beams. The pump pulses (centered at 785 nm, 100 fs) was produced by a collinear optical parametric amplifier (TOPAS-C, Light Conversion). A second TOPAS was used to generate 1350 nm pulses, which were focused on a CaF_2_ crystal to generate broadband white light probe. The single-color femtosecond pump probe measurement used the following setup^46^. An amplified femtosecond laser system (Pharos, Light conversion) operating at 1030 nm and delivering pulses of 200 fs at a repetition rate of 1 kHz pumps two non-collinear optical parametric amplifiers (NOPAs, Orpheus-N, Light Conversion). One of them was used to generate pump pulses centered at 785 nm with a pulse duration of 100 fs. The second NOPA was used to generate probe pulses at 830 nm and 870 nm, respectively, for differential transmission measurements. In both setups, pump and probe pulses were almost collinear. The probe was time delayed with respect to the pump by a mechanical delay stage. The laser beams and the cavity were oriented so that they were at the angle of 30° in respect of each other. At that angle the cavity mode is in resonant with the B850 exciton. Polarization of the probe pulse was set to TE mode. The measurements were performed at room temperature. The signals were recorded using low-energy (8.5 μJ/cm^2^/pulse) pumping pulses to avoid spurious effects and exciton−exciton annihilation in LH2 film. The data presented in this work are reported in terms of -log_10_(*T/T*_*0*_) to connect to the majority of transient absorption studies conducted outside cavities, where *T* is the transmission measured with the pump pulse present and *T*_*0*_ is the transmission without the pump pulse. The data were analyzed and fitted with a rate-based kinetic model, formulated in MATLAB using the Nelder–Mead–Simplex method and the sum of least squares to minimize the error^38,39^.

### Coupled oscillator model

The observed polariton branches in the cavity sample were fitted with a 3-by-3 coupled oscillator model described by the following Hamiltonian:

$$\left(\begin{array}{ccc}{E}_{{{\mbox{Cav}}}} & {V}_{1} & {V}_{2}\\ {V}_{1} & {E{{\mbox{x}}}}_{{{\mbox{B}}}850} & 0\\ {V}_{2} & 0 & {E{{\mbox{x}}}}_{{{\mbox{B}}}800}\end{array}\right)\left(\begin{array}{c}\alpha \\ \beta \\ \gamma \end{array}\right)=E\left(\begin{array}{c}\alpha \\ \beta \\ \gamma \end{array}\right)$$

where *α*, *β*, and *γ* are the mixing coefficients of the eigenvectors of the strongly coupled systems. The Hopfield coefficients in each polariton are given as |*α* | ^2^, |*β* | ^2^, and |*γ* | ^2^. *E*_Cav_ is the energy of cavity mode, and *E*x_B850_ and *E*x_B800_ the exciton energies of B850 band and B800 band. *E* represents the eigenvalues corresponding to the energy of the formed polaritons. The cavity mode energy is given as^47^ ${E}_{{{\mbox{Cav}}}}(\theta)={E}_{0}/\sqrt{1-{({{{\sin}}\,\theta }/{{n}_{{{\mbox{eff}}}}})}^{2}}$. Here, *θ* is the angle of incidence and *E*_*0*_ is the cavity energy at *θ* = 0°. *n*_eff_ is the effective refractive index. The coupling strength *V* is related to the Rabi splitting *ħΩ*_R_, when *E*_Cav_ = *E*x, it is given by $V=\hslash {\varOmega }_{{{\mbox{R}}}}/2$. Here, *V*_*1*_, *V*_*2*_, and *n*_eff_ are obtained by globally fitting the dispersion curves to the eigenstates of the Hamiltonian, using the Trust Region Reflective algorithm.

## Supplementary information

Supplementary Information

## Data Availability

The data that support the findings of this study are available from the corresponding author upon request.
