# Supplementary material for: Optical cavity-mediated exciton dynamics in photosynthetic light harvesting 2 complexes
Source: Nat Commun. 2022 Nov 11;13:6864. doi: 10.1038/s41467-022-34613-x (PMC9652305; doi:10.1038/s41467-022-34613-x)
Supplement: Supplementary file 1 — Supplementary Information [file 41467_2022_34613_MOESM1_ESM.pdf]

Supplementary Information

Optical cavity-mediated exciton dynamics in photosynthetic light harvesting 2 complexes

*Fan Wu[1], Daniel Finkelstein-Shapiro[1,2], Mao Wang[3], Ilmari Rosenkampff,[1] Arkady Yartsev[1], Torbjörn Pascher[1], Tu C. Nguyen- Phan[4], Richard Cogdell[4], Karl Börjesson[3], Tönu Pullerits[1]**

[1] Division of Chemical Physics and NanoLund, Lund University, Sweden; [2] Instituto de Química, Universidad Nacional Autónoma de México, Mexico;[3] Department of Chemistry and Molecular Biology, University of Gothenburg, Sweden; [4] Institute of Molecular, Cell, and Systems Biology, University of Glasgow, UK

E-mail: tonu.pullerits@chemphys.lu.se

Contents

Supplementary Figure 1: Determination of Q-factor.

Supplementary Figure 2: Angle-resolved reflection and transmission spectra of the strongly coupled LH2 containing cavity sample.

Supplementary Note: Transient absorption result of bare LH2 film.

Supplementary Figure 3: Pump excitation intensity dependent TA of bare LH2 film.

Supplementary Figure 4: Broadband pump probe spectra of bare LH2 film, strongly coupled LH2 cavity sample and weakly coupled LH2 cavity sample; steady Steady-state transmission spectrum of weakly coupled LH2 sample.

Supplementary Note 2: Evaluation of the non-specific photoexcitation induced effects in ultrafast pump probe measurements.

Supplementary Figure 5: Transmission spectrum change from non-specific photoexcitation induced effects in microcavity.

Supplementary Figure 6: Evaluation of the uncertainties of the fitted parameters of the pump probe kinetics of the strongly coupled sample.

Supplementary Figure 7: Comparison of the normalized pump probe kinetics of bare LH2 film and the strongly coupled cavity sample.

Supplementary References

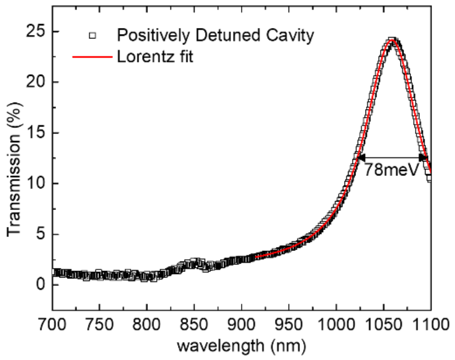

Supplementary Figure 1 Determination of Q-factor. Transmission spectrum of a negatively detuned LH2 containing cavity. The FWHM of the peak is approximately 78 meV. The energy of the transition is approximately 1.17 eV. Q=1.17eV/0.078 eV≈15.

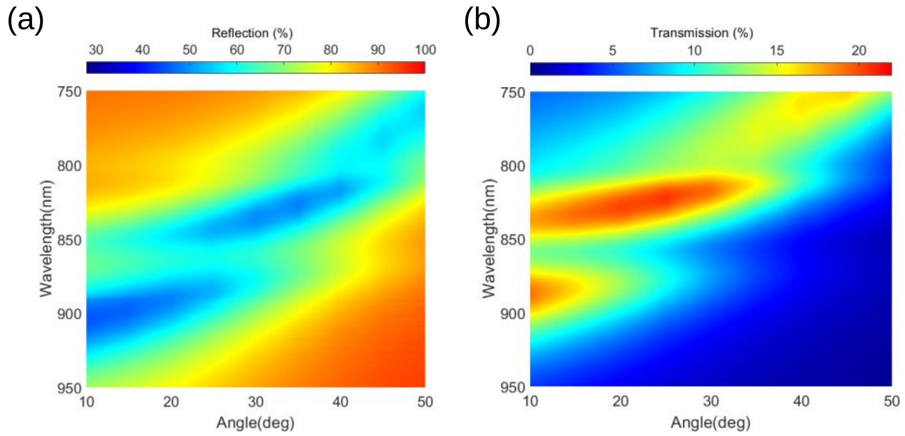

Supplementary Figure 2 Angle-resolved (a)reflection and (b) transmission spectra of the strongly coupled LH2 containing cavity sample.

Supplementary Note 1: Transient absorption (TA) result of bare LH2 film

We note that the lifetime of B850 band in bare LH2 film is much shorter than that of the LH2 solution (~1ns) which has been studied extensively. A pump intensity dependent measurement (see Figure S3) was performed, and the TA kinetic signal at intensities of 4.2 and 8.5 μJ/pulse/cm^2 overlapped with each other after normalization, showing that exciton-exciton annihilation cannot explain the shortening.

In order to quantify the processes in bare LH2 film, a rate-based kinetic model was employed to fit the data. The rate equations can be written as:

$$\frac{d[B800^*]}{dt} = -k_{B800toB850s}[B800^*]$$

$$\frac{d[B850\_1^*]}{dt} = k_{B800toB850s}[B800^*] - k_{B850\_1}[B850\_1^*]$$

$$\frac{d[B850\_2^*]}{dt} = k_{B800toB850s}[B800^*] - k_{B850\_2}[B850\_2^*]$$

$Where\ k_{B800toB850s}$ is the energy transfer rate from the excited B800 band to B850 bands, and k_{B850_1} and k_{B850_2} are the two rates corresponding to the excited B850 bands of different LH2s decaying to the ground state. $k_{B800toB850s}$ is determined to be 1.09 ps^{-1} as a result of the modelling, which coincides with the value of the energy transfer rate of B800 to B850 in LH2 solution. To get better fitting of the experimental result, two decaying routes of the B850 bands from different LH2s to ground state were considered in the model, which are determined to be 0.021 ps^{-1} and 0.0071 ps^{-1}, respectively. These rates are much faster than the decaying of B850 band in LH2 solution (0.001 ps^{-1}). Similar lifetime shortening upon solidification of the materials has been monitored in other organic semiconductor materials[1,2], which was ascribed to the formation of new solid-state band. Besides, it is also likely to be induced by the protein structural deformation in LH2 film[3].

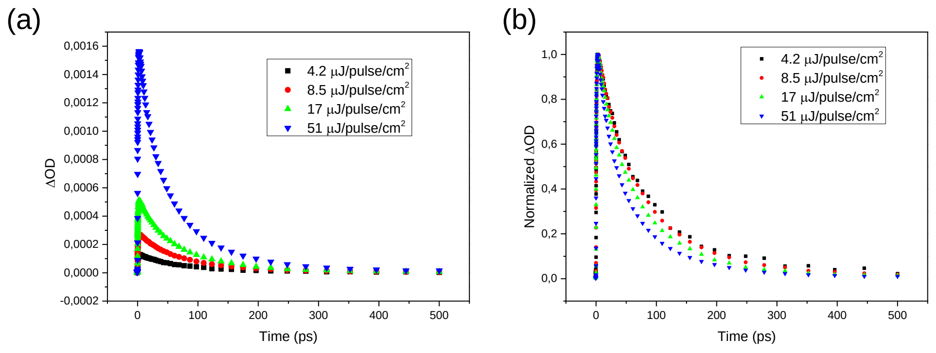

Supplementary Figure 3 Pump excitation intensity dependent TA of bare LH2 film (a) before and (b) after normalization. The near perfect overlap between the kinetic traces at intensities of 4.2 and 8.5 μJ/pulse/cm^2 indicates that below 8.5 μJ/pulse/cm^2 the contribution of exciton-exciton annihilation is negligible.

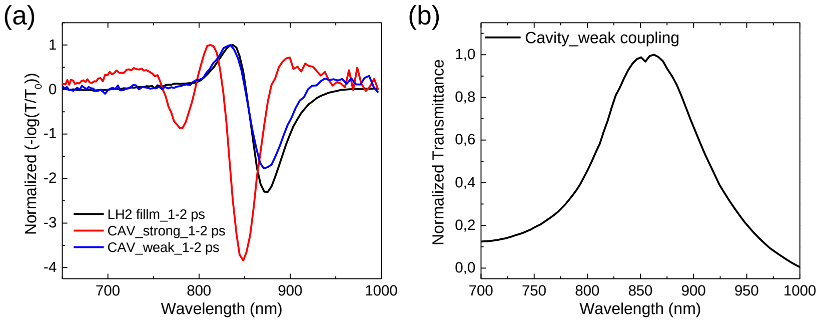

Supplementary Figure 4 (a) Broadband pump probe spectra of bare LH2 film (black), strongly coupled cavity sample (red) and weakly coupled cavity sample (blue) at 1~2 ps; note: the weakly coupled cavity sample was prepared the same way as the strongly coupled cavity except the concentration of LH2 used for spin coating was much lower than that for the strongly coupled cavity sample; (b) Steady-state transmission spectra of the weakly coupled cavity sample, which shows negligible energy splitting.

Supplementary Note 2: Evaluation of the non-specific photoexcitation induced effects in ultrafast pump probe measurements

Here the non-specific photoexcitation induced effects are mainly evaluated from the pump-induced thermal effect and the Rabi contraction which is resulting from the pump-induced reduction of the ground state population. As for the thermal effect induced by the pump excitation, the temperature change of the lattice is estimated as follows. First, the average number of excitations per LH2 ring is calculated to be 0.07 per pulse. This is calculated from knowing the absorbance, the extinction coefficient, thickness of the LH2 film and the pump intensity. Then according to the pump photon energy and the number of degrees of freedom calculated from the non-hydrogen atoms in LH2, and the ratio of LH2 to PVA, a temperature change of 0.1 K is estimated. This induces change in the thickness of the LH2 film by ~ 0.0004% [4] and change in refractive indexes of the LH2 films by ~ 10^{-6}.[5] This temperature change also coincides with the thermal effect induced by similar photo excitation reported previously[6,7]. The pump-induced reduction of the ground state population of chlorophyll molecules is estimated to be 0.0025, based on the excitation per LH2 ring and the number of chlorophyll molecules per LH2 ring.

To figure out the possible contributions of these non-specific photoexcitation effects on the pump probe signal, we employed the 3-by-3 coupled oscillator model as described in the main text to calculate the microcavity optical properties. As input to the model, the absorption spectrum of the strongly coupled cavity sample at 30° was fitted using a series of Lorentzian functions, and the parameters of the model are optimized by non-linear least squares method. The fitted absorption spectrum is shown in Supplementary Figure 5a, which is in a good agreement with the experimentally measured spectrum. Based on the current model, the transmission spectrum change induced by all the non-specific photoexcitation effects is calculated (see Supplementary Figure 5b), taking the following parameters into consideration: i) ground state bleaching of 0.0025; ii) increase of the LH2 film thickness of ~ 0.0004 %; iii) refractive index decrease in LH2 film by ~ 10^{-6}. Comparing the spectral shape of the calculated spectrum with the measured pump probe spectra of the strongly coupled cavity, we can see a clear deviation, especially in the red region where opposite sign is presented.

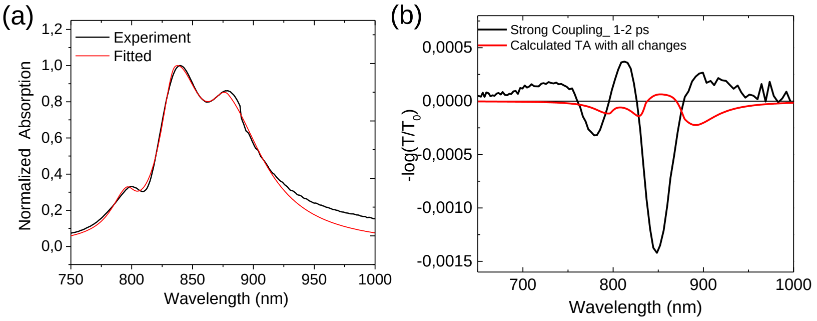

Supplementary Figure 5 (a) Comparison of fitted absorption spectrum (red) using 3-by-3 coupled oscillator model and experimentally measured absorption spectrum (black) of the strongly coupled cavity sample at 30°; (b) comparison of the calculated transmission change spectrum (red) from all the non-specific photoexcitation induced effects and the measured pump probe spectrum (black) of the strongly coupled cavity sample.

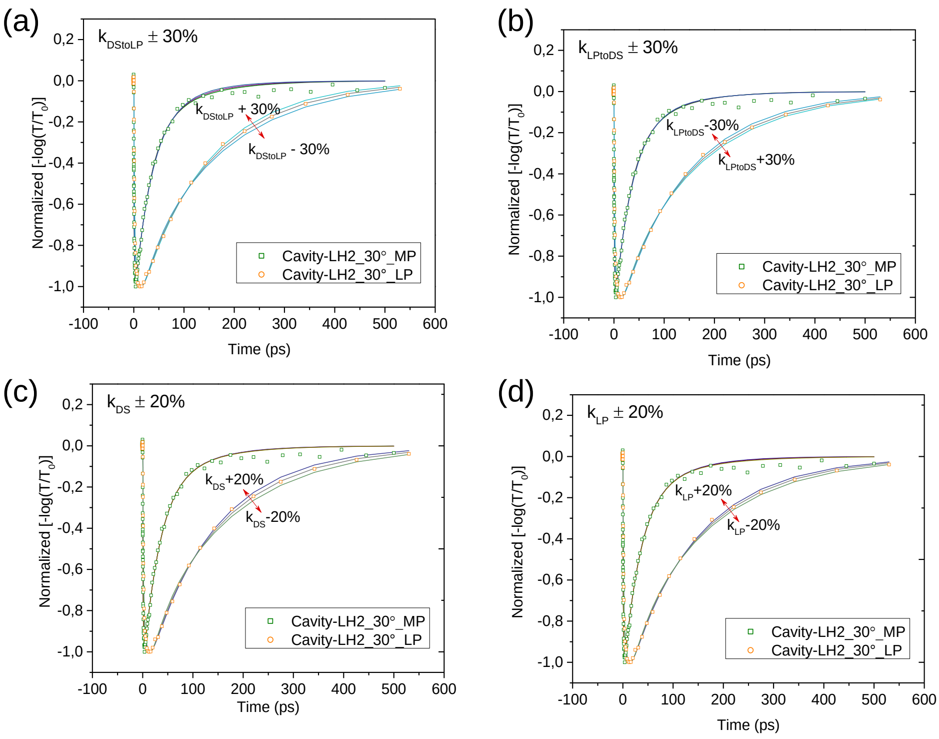

Supplementary Figure 6 Fitting of pump probe kinetics of both MP and LP states of the strongly coupled cavity sample at 30° with uncertainties of (a) k_{DStoLP},(b) k_{DStoLP},(c) k_{DStoLP} and (d) k_{DStoLP}, respectively. Note that uncertainties of the fitted parameters were evaluated by varying the parameters one-by-one within a certain range.

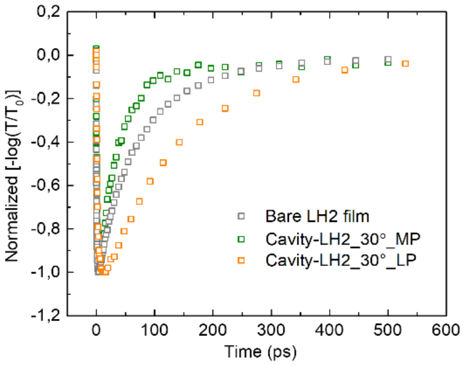

Supplementary Figure 7 Comparison of the normalized pump probe kinetics of bare LH2 film (grey), the MP (green) and LP (orange) states of the strongly coupled cavity sample at 30°.

Supplementary References

1. Im, C., Kang, S., Choi, J. & An, J. Comparing Donor- and Acceptor-Originated Exciton Dynamics. **13**, 1770 (2021).

2. Wang, R. *et al.* Charge Separation from an Intra-Moiety Intermediate State in the High-Performance PM6:Y6 Organic Photovoltaic Blend. *J. Am. Chem. Soc.* **142**, 12751–12759 (2020).

3. Chen, X. H. *et al.* Protein structural deformation induced lifetime shortening of photosynthetic bacteria light-harvesting complex LH2 excited state. *Biophys. J.* **88**, 4262–4273 (2005).

4. Tilton, R. F., Dewan, J. C. & Petsko, G. A. Effects of temperature on protein structure and dynamics: x-ray crystallographic studies of the protein ribonuclease-A at nine different temperatures from 98 to 320K. *Biochemistry* **31**, 2469–2481 (1992).

5. Waxler, R. M. & Cleek, G. W. The Effect of Temperature and Pressure on the Refractive Index of Some Oxide Glasses. *J. Res. Natl. Bur. Stand. Sect. A, Phys. Chem.* **77A**, 755–763 (1973).

6. Liu, B., Menon, V. M. & Sfeir, M. Y. The Role of Long-Lived Excitons in the Dynamics of Strongly Coupled Molecular Polaritons. *ACS Photonics* **7**, 2292–2301 (2020).

7. Liu, B., Menon, V. M. & Sfeir, M. Y. Ultrafast thermal modification of strong coupling in an organic microcavity. *APL Photonics* **6**, 16103 (2021).
